# Overexpression of microRNA-145 inhibits tumorigenesis through autophagy in chemotherapy and radiation resistant neuroblastoma cells

**DOI:** 10.18632/oncoscience.496

**Published:** 2020-02-01

**Authors:** Kwang Woon Kim, Jingbo Qiao, Julia Y Kim, Kyungho Park, Dai H Chung

**Affiliations:** ^1^ UT Southwestern Medical Center, Department of Surgery, Dallas, TX, USA

**Keywords:** chemotherapy, radiotherapy, microRNA-145, LC3 I&II, neuroblastoma

## Abstract

MicroRNA-145 (miR-145) plays a suppressive role in the process of tumorigenesis and an important role in induction of autophagy. However, the exact role of miR-145 in therapeutically resistant neuroblastoma cells remain elusive. Herein, we sought to evaluate the effects of miR-145 overexpression in chemo‑ and radiation-resistant neuroblastoma cells. We hypothesized that miR-145 affects the aggressiveness of resistant cells by enhancing autophagy. We established Cisplatin-resistant (CDDP-R), Vincristine-resistant (Vin-R), and radiation-resistant (Rad-R) neuroblastoma cells and found that miR-145 expression was significantly decreased in the resistant cells compared to the parental cells. Exogenously expression of miR-145 inhibited oncogenic properties such as proliferation, clonogenicity, anchorage-independent growth, cell migration, and tubule formation in the resistant cells. In addition, we also found that an autophagy protein marker, LC3, was only minimally expressed in the resistant cells. In particular, when miR-145 was overexpressed in the resistant cells, LC3 I and II were expressed and an increased punctate fluorescence of LC3 protein was found indicating the induction of autophagy. Taken together, our data suggests that miR-145 inhibits tumorigenesis and aggressiveness via modulation of autophagy in neuroblastoma.

## INTRODUCTION

Neuroblastoma, a malignant embryonal tumor that can occur anywhere along the sympathetic nervous system, usually arises in the adrenal glands. It remains the most common extracranial childhood tumor and accounts for 15% of all childhood cancer deaths [1]. The biological behavior of neuroblastoma is extensively heterogeneous, ranging from spontaneous regression to rapid progression despite aggressive, multimodal therapy [1].

MicroRNAs (miRNAs) were first discovered in C. elegans as a class of short, noncoding RNAs that contain around 22 nucleotides. miRNA functions as a negative regulator of gene expression by inducing mRNA degradation or by repression of translation repression by targeting the 3'-untranslated region (3'-UTR) of miRNAs [2]. miRNAs play an important role in several aspects of tumor biological processes such as proliferation, developmental timing, growth control, differentiation, invasion, metastasis, and angiogenesis [3]. Among miRNAs, miR-145 was first discovered based on its homology to a verified mouse miRNA [4,5]. Reduced expression of miR-145 was first reported in colonic adenocarcinoma, suggesting a possible role of miR-145 in the early stage of colorectal cancer [6]. Following the initial discovery of miR-145, several studies have demonstrated deregulation of miR-145 acting as a tumor suppressor in various human malignancies, including neuroblastoma [7]. Recent studies indicate that miR-145 is downregulated in the primary central nervous system of lymphoma and glioblastoma, implicating its potential role in nervous system tumors [8, 9]. However, the exact function and downstream targets of miR-145 in neuroblastoma are still an enigma.

Autophagy is a self-digestive process that regulates a highly conserved catabolic process under various stress conditions in cells. The degradation of long-lived proteins or cellular components, such as damaged mitochondria, occurs via autophagy. It is characterized by the formation of cytoplasmic double-membrane vacuoles, named autophagosomes, which fuse with lysosomes [10-12]. This process has recently been identified as a physiological response to miRNA modulation [13]. In particular, miR-145 regulated autophagy has been shown to potentially regulate proliferation, clonogenicity, anchorage-independent growth, cell migration, and tubule formation by vascular endothelial cells [13].

Here we report, for the first time, that miR-145 is downregulated in Cisplatin-resistant (CDDP-R), Vincristine-resistant (Vin-R), and radiation-resistant (Rad-R) neuroblastoma cells. We also report that overexpression of miR-145 in these resistant cells inhibits proliferation, clonogenicity, anchorage-independent growth, cell migration, and tubule formation in vascular endothelial cells, and that this may occur partially through induction of autophagy. Our findings suggest the potential use of miR-145 as a novel therapeutic strategy in drug- and radiation-resistant neuroblastoma. Future studies are needed to elucidate the exact targets of miR-145 and the precise mechanisms by which miR-145 acts in the resistant cells.

## RESULTS

### miR-145 expression in chemo‑ and radiation-resistant neuroblastoma cells.

**Figure 1:**
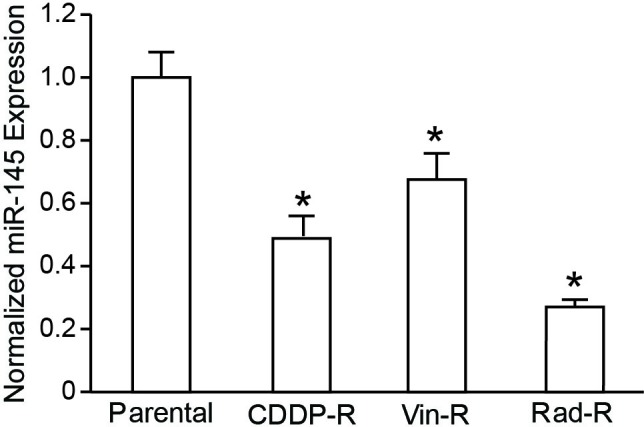
QRT-PCR analysis of miR-145 in CDDP-R, Vin-R, and Rad-R human neuroblastoma cells Expression of miR-145 was normalized to expression of miR-145 in parental BE(2)-C cells. Expression levels of miRNA are shown as relative ratios with respect to the SNORD61 expression level and calculated by the 2‐^ΔΔC(t)^ method. Data are the mean ± SEM of three independent RT-PCRs (* *P* < 0.05 vs. Parental).

To study the molecular mechanisms of chemotherapy and radiation therapy resistance in neuroblastoma, CDDP-R, Vin-R and Rad-R cells were used. Briefly, BE(2)-C cells were treated with CDDP, vincristine, and radiation at 50% survival dose. This process of generating resistant cells continued for six months and these cells obtained more aggressive characteristics than parental cells [14]. Given that miRNAs play a tumor suppressor role in various cancers, and miR-145 is expressed specifically in neuroblastoma cells [7], we sought to examine the expression of miR-145 in our resistant cells. We performed qRT-PCR and found that miR-145 expression was significantly lower in all resistant cells, down to 48.7% in CDDP-R, 67.5% in Vin-R, and 28.3% in Rad-R, compared to miR-145 expression in parental cells (Figure 1). This result implies that the resistant cells may exhibit a tumorigenesis potential due to the decreased level of miR-145.

### miR-145 overexpression decreased cell growth and colony formation in chemo- and radiation-resistant neuroblastoma cells.

**Figure 2:**
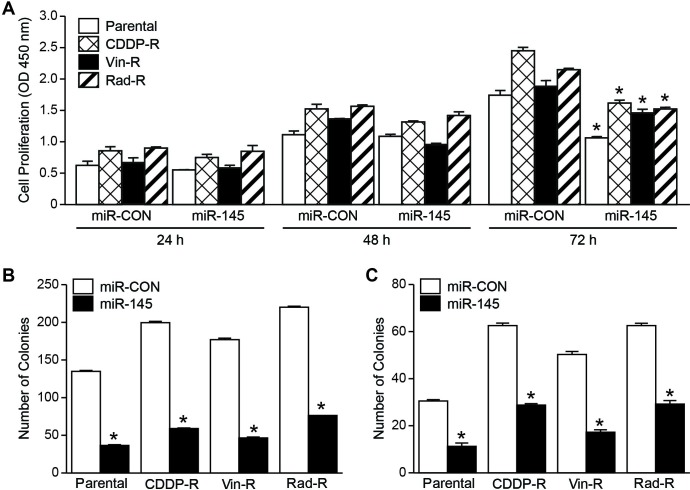
Overexpressed miR-145 decreases proliferation, clonogenic colonies, and soft agar colonies in neuroblastoma cells. (A) Cell proliferation was measured in parental, CDDP‐R, Vin‐R, and Rad‐R BE(2)‐C cells transfected with miR-CON and miR-145. (B) Cell clonogenic assay was performed and quantified. (C) Anchorage-independent growth was assessed by soft agar colony assay in parental, CDDP‐R, Vin‐R, and Rad‐R BE(2)‐C cells transfected with miR‐CON and miR-145 plasmid. Data are the mean ± SEM of three separate experiments (* *P*< 0.05 vs. miR-CON).

Given the tumor suppressor role of miR-145 in neuroblastoma, we sought to investigate whether overexpression of miR-145 inhibits proliferation of CDDP-R, Vin-R, and Rad-R BE(2)-C cells by measuring cell viability with the Cell Counting Kit-8 kit. As expected, we observed that overexpression of pCMV-miR-145 (miR-145) in parental cells reduced cell proliferation compared to pCMV-miR vector (miR-CON). Furthermore, although CDDP-R, Vin-R, and Rad-R BE(2)-C cells showed endogenously low expression of miR-145, forced expression of miR-145 in these resistant BE(2)-C cells led to significantly decreased cell proliferation after 72 h (Figure. 2A). Next, we performed a colony formation assay, which is an essential assay in determining the ability of each cell to undergo “unlimited” division [15]. Cells were plated in a 6-well plate and cultured for 10 days. We found that overexpression of miR-145 in the CDDP-R, Vin-R, and Rad-R BE(2)-C cells significantly reduced the number of colonies to 29.3%, 26%, and 34.3%, respectively, in comparison to miR-CON, indicating a tumor suppressive property of miR-145 (Figure 2B). Furthermore, we performed a soft agar colony formation assay in order to examine the anchorage-independent growth ability, one of the hallmarks of cell transformation. This method has been accepted as *in vitro* assay for detecting cell malignancy and correlates with tumorigenicity *in vivo* [16]. CDDP-R, Vin-R, and Rad-R BE(2)-C cells transfected with miR-145 or miR-CON were plated in soft agar and cultured for 2 weeks as described previously [17]. Our results showed that overexpression of miR-145 significantly decreased anchorage-independent growth of resistant cells, and the number of colonies was decreased to 45.7% in CDDP-R, 34.4% in Vin-R, and 47.1% in Rad-R, in comparison to miR-CON (Figure 2C). Our findings further indicate that overexpression of miR-145 inhibits a transformation property of CDDP-R, Vin-R, and Rad-R neuroblastoma cells.

### Exogenous expression of miR-145 inhibited angiogenesis and cell migration in chemo- and radiation-resistant neuroblastoma cells.

**Figure 3:**
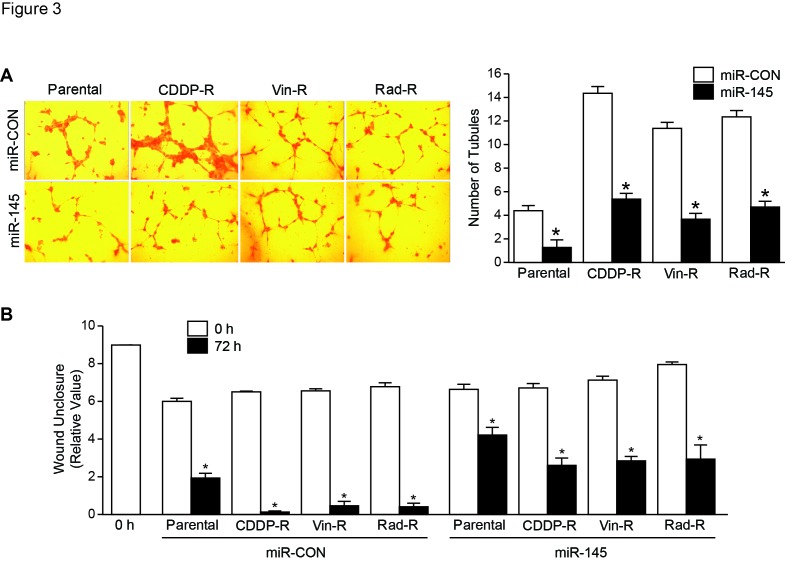
Overexpressed miR-145 decreases formation of capillary-like tubes by HUVECs and migration in neuroblastoma cells. (A) HUVECs were plated on Matrigel and incubated with cell culture media from parental, CDDP-R, Vin-R, and Rad-R cells transfected with miR-CON and miR-145 plasmid for 48 h. Representative tubule images were shown (X200). Values shown are mean ± SEM of three separate experiments (* *P*< 0.05 vs. miR-CON). (B) Cells were plated for 24 h, then scratches were made. Wound closure was measured from microscopic images at 0 h and 72 h. Values shown are mean ± SEM of three separate experiments (* *P*< 0.05 vs. miR-CON).

To examine whether overexpression of miR-145 affects tubule formation and, in turn, stimulates angiogenesis of these resistant cells *in vitro*, we cultured human umbilical vein endothelial cells (HUVECs) with conditioned media using cell culture supernatant from both parental and resistant cells. We observed that the number of tubules formed by HUVECs grown in media from miR-145-overexpressed parent cells was decreased compared with that from miR-CON. As expected, we found that tubule formation was significantly decreased when HUVECs were grown in media from CDDP-R, Vin-R, and Rad-R BE(2)-C cells transfected with miR-145, down to 37.2%, 32.4%, and 37.8%, respectively, compared with that from miR-CON. This suggests that overexpression of miR-145 can inhibit angiogenesis in the resistant cells to levels similar in parental cells (Figure 3A). Additionally, we sought to determine the effects of miR-145 on cell migration by performing a wound healing assay. Wound closure was measured microscopically and determined by the relative values of the remaining wound space. Overexpression of miR-145 significantly decreased cell migration at 72 h after wounding in these resistant cells when compared to miR-CON, and was similar to parental cells (Figure 3B). These results suggest that overexpression of miR-145 can result in decreased angiogenesis and tumor metastasis as determined by tubule formation and cell migration assays.

### Overexpression of miR-145 induced autophagy in chemo- and radiation-resistant neuroblastoma cells.

**Figure 4:**
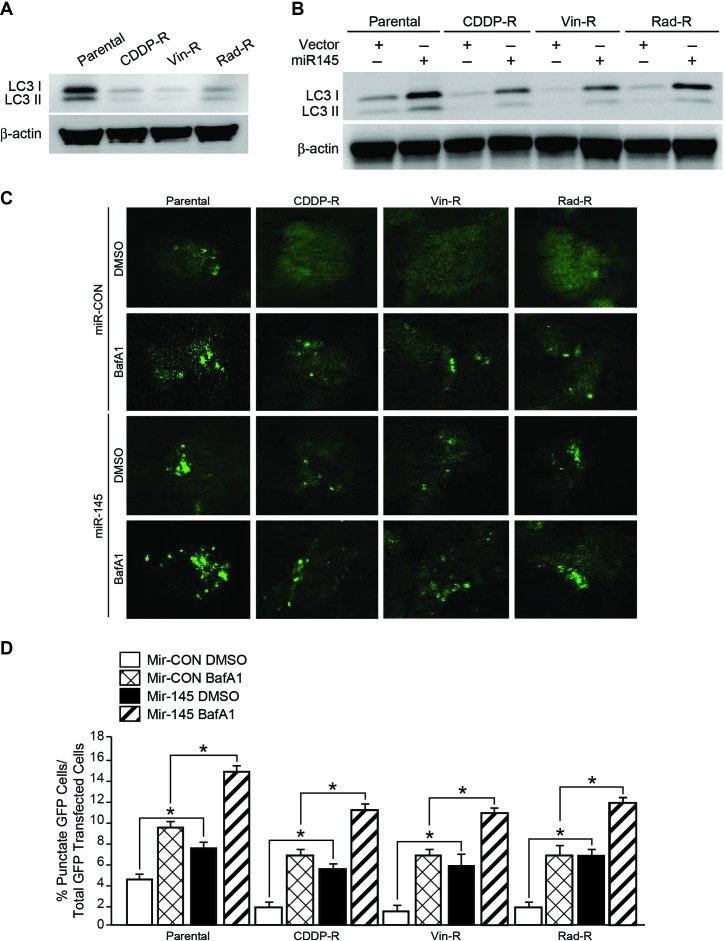
Overexpressed miR-145 increases LC3 I & II protein levels in neuroblastoma cells. (A) LC3 protein was detected with immunoblotting in cells. β-actin served as a loading control. (B) Parental, CDDP-R, Vin-R, and Rad-R BE(2)-C cells transfected with miR-CON and miR-145 plasmid. LC3 I & II protein level was detected. β-actin served as a protein loading control. (C) Parental, CDDP-R, Vin-R, and Rad-R BE(2)-C cells co-transfected with miR-CON or miR-145 plasmid with eGFP-LC3 plasmid, treated with or without BafA1 (200 nM) for 24 h and then assessed for autophagy after 48 h transfection. Representative fluorescence confocal microscopic images were taken. (D) Cells with GFP-LC3 puncta were quantified as percentage of total GFP cells for each experiment (n = 3). At least 20 cells were counted in each individual experiment. Values shown are mean ± SEM of three separate experiments (* *P*< 0.05 vs. miR-CON).

A recent study showed that miR-145 enhances autophagy, which was indicated by increased levels of autophagosome marker, LC3 I and LC3 II [18]. To determine whether resistant cells have endogenously changed this autophagic property, we performed western blot analysis to measure the protein level of LC3 I and LC3 II. Interestingly, all resistant cells expressed a significantly lower level of both LC3 I and LC3 II, when compared to parental cells (Figure 4A). We next wanted to examine the ectopic expression effect of miR-145 on autophagy in resistant cells. We found that miR-145 reversed the endogenously decreased LC3 level, and that overexpression of miR-145 in CDDP-R, Vin-R, and Rad-R BE(2)-C neuroblastoma cells led to increased expressions of LC3 I and LC3 II (Figure 4B).

To further visualize the formation of autophagosomes, parental, CDDP-R, Vin-R, and Rad-R BE(2)-C cells were transfected with EGFP-LC3 plasmids in addition to miR-CON or miR-145 plasmid. Then an autophagy flux assay was performed in cells treated with or without bafilomycin A1 (BafA1), the vacuolar H+−ATPase (V-ATPase) inhibitor which blocks the fusion of autophagosome with lysosome. Diffuse cytoplasmic localization of EGFP-LC3 was observed in resistant cells transfected with miR-CON, whereas resistant cells overexpressing miR-145 showed significantly increased number of punctate fluorescence of LC3 protein at 48 h post-transfection, similar results were observed in parental cells (Figure 4C). In particular, the punctuated EGFP-LC3 protein was significantly accumulated in cells under treatment with BafA1. miR-145 increased the punctuated EGFP-LC3 in resistant cells when compared with resistant cells transfected with miR-CON (Figure 4C); these effects of miR-145 were assessed quantitatively by determining the percentage of punctate GFP cells over total GFP transfected cells (Figure 4D). Taken together, these results suggest that miR-145 induced autophagy in these resistant cells.

## DISCUSSION

Neuroblastoma is the most common extracranial solid tumor in childhood and demonstrates high cellular heterogeneity, which leads to a wide range of clinical presentations and various responses to existing treatments. Furthermore, therapeutic chemo-/radiation-resistance induced during the clinical courses is one of the major obstacles to the treatment of patients with an advanced-stage, high-risk neuroblastoma. Recently, several studies revealed that miRNAs are involved in drug resistance of tumor cells that have been treated with chemotherapy drugs [19].

miRNA have been shown to act as tumor suppressors as well as oncogenes in various cancer cells including lung, brain, breast, and liver cancer. Specifically, as it pertains to neuroblastoma, miR-17-5p-92 operates in an oncogenic fashion, whereas miR-34a exhibits several tumor suppressor capabilities [19]. In addition, miRNA profiling has been increasingly indispensable in cancer prognosis and diagnosis. miRNAs function at the post-transcriptional level to degrade or repress the translation of mRNA [20]. They have been proven to regulate various cellular processes and thus, dysregulation of miRNAs has been associated with the pathogenesis of cancer [21]. miRNAs regulate roughly 50% of the human genome and control numerous molecular pathways of cancer [22]. Due to the extensive influence of miRNAs on the proliferation, apoptosis, and maintenance of various cancer cell lines, targeting miRNAs as a potential therapeutic strategy has been of growing interest [23].

Chemotherapy and radiotherapy have been the main approach for the treatment of high-risk neuroblastoma, but radio- and chemo-resistance has proven to be a major obstacle to effective treatment [24, 25]. Therefore, identification of new therapeutic strategies to combat radio- and chemo-resistance has long been a goal in cancer therapy efforts. As mentioned, over the past decade it has become clear that miRNA expression is dysregulated in human malignancies [3]. Recently, it was found that the disorder of several miRNAs could lead to chemo- and radio-resistance, by preventing oncogenic or tumor-suppressive functions, in virtually all forms of cancer including neuroblastoma [26-29]. Specifically, among these miRNAs, the miR-145 plays an important role in many pathological and physiological processes. Low expression of miR-145 was associated with advanced clinical stage, lymph node metastasis, larger tumor diameter, and shorter disease-free survival in prostate cancer patients and small cell carcinoma of the cervix [30]. miR-145 has also demonstrated tumor suppressor properties such as tumor growth, angiogenesis, and metastasis in various cancers, including neuroblastoma and others [7, 31, 32]. In particular, a recent report showed that miR-145 was involved in regulation of autophagy in cancer [33].

In the present study, we found that miR-145 is downregulated in CDDP-R, Vin-R, and Rad-R BE(2)-C cells compared with parent BE(2)-C cells. We also found that exogenous expression of miR-145 in the resistant cells downregulated neuroblastoma growth, angiogenesis, and cell migration. In addition, LC3, an autophagosome marker, was significantly downregulated in these resistant cells, and overexpression of miR-145 in the cells induces the expressions of LC3 I and II as well as increased EGFP-LC3 puncta, suggesting that the correlation between miR-145 and autophagy is involved in the regulation of treatment-resistant neuroblastoma. Further investigation is needed to identify the exact mRNA targets of miR-145 and the precise mechanisms by which miR-145 acts in the resistant cells. Current results revealed that miR-145 overexpression in chemo-/radio-resistant cells attenuated proliferation, clonogenic growth, soft agar growth, angiogenesis, and cell migration, demonstrating that miR-145 plays a tumor suppressor role. These findings were consistent with other studies [7, 13, 31] where miR-145 expression resulted in the inhibition of tumorigenesis.

In conclusion, the current study identified the fact that miR-145 mediates autophagy in drug/radiation resistance, which might, in turn, inhibit neuroblastoma tumorigenesis. To our knowledge, this is the first report of inhibitory oncogenic properties occurring in correlation with miR-145 and a key autophagy marker LC3, in resistant neuroblastoma cells. Future studies about biological interaction between the effectors of miR-145 and autophagy are needed. Moreover, miR-145 may be a potential therapeutic strategy to induce autophagy-mediated neuroblastoma cell death in drug/radiation resistance neuroblastoma. Therefore, understanding the relationship between miR-145 and the autophagy process, and their influences on neuroblastoma tumorigenesis will contribute to improvements of overall outcomes for neuroblastoma patients with the aggressive, refractory form of this disease.

## MATERIALS AND METHODS

### Cell culture, reagents and antibodies

Neuroblastoma BE(2)-C cells were cultured in RPMI 1640 (Mediatech, Cat No. 10-040-CV) supplemented with 10% fetal bovine serum (Sigma-Aldrich, Cat No. F2442) at 37°C and humidified 5% CO_2_. HUVECs obtained from Dr. M. Freeman (Vanderbilt University Medical Center) were cultured in EMM-2 supplemented with Growth Factors (EGM-2 SingleQuot kit, Lonza, Cat No. CC-4176) at 37°C and humidified 5% CO_2_. The miR-CON, pCMV-miR-145 plasmid was obtained from Dr. Yingjie Yu (Wayne State University School of Medicine). Cisplatin (CDDP) was purchase from Sigma-Aldrich (Cat No 15663-27-1), and Vincristine was purchased from Sigma-Aldrich (Cat No. V8879). The primary antibodies used include LC3-1&II (MBL, PD014), ꞵ-actin antibody was from Sigma-Aldrich (1:5000, A2066) and Bafilomycin A1 (BafA1) was purchased from LC Laboratories (B-1080).

### Generation of CDDP-resistant, vincristine-resistant and radiation-resistant neuroblastoma cells

BE(2)-C (3x10^5^) cells were plated onto 100 mm x 20 mm plates and cultured as described above. After incubation for 24 h, the cells were treated with CDDP, vincristine and radiation. The doses at which 50% of cell survival was inhibited by CDDP and vincristine were 5 µM and 0.5 µM, respectively. The radiation dose at which 50% of cell survival inhibited was 10G. A ^137^Cs irradiator was irradiated with 10G (J.L. Shepherd and Associates, Glendale, CA) at room temperature (dose rate 1.8 Gy/min). After incubation for 7 days, the media was removed, and the surviving cells were allowed to recover for an additional 25 days. This development period was carried out for approximately 6 months, after which time resistance was reassessed by treating the resistance cells with the previously calculated 50% survival dose of CDDP, radiation, and vincristine and confirming cell survival.

### Quantitative real-time PCR (QRT-PCR)

Total RNA was isolated using the RNAqueous™ (Life Technologies). Isolated RNA (1 µg) was used to synthesize cDNA using the High-Capacity cDNA Reverse Transcription Kit (Life Technologies). QRT-PCR was performed in the CFX96™ Real-Time PCR Detection Systems using SsoFast™ EvaGreen Supermix (Bio-Rad). MicroRNA-145 was detected using miScript primer (forward primer, Qiagen, MS0000312220) and miScript universal primer (reverse primer, Qiagen). Gene expression was normalized to SNORD61.

### Cell proliferation analysis

Cells (5000 cells/well) were seeded onto 96-well plates in RPMI 1640 culture media containing 10% FBS and grown for up to 4 days. Cell viability was assessed using Cell Counting Kit-8 kit daily. The values corresponding to the number of viable cells were read at OD450 with the FlexStation 3 Microplate Reader (Molecular Devices, Sunnyvale, CA).

### Clonogenic assay

Cells were trypsinized and resuspended in RPMI 1640 media with 10% FBS and plated in 6-well plates (1,000 cells/well) and incubated for 10 days. Colonies were fixed and stained with 0.005% crystal violet in 70% methanol for overnight. The colony images were taken with Bio-Rad Gel Doc XR+ Imaging System, and quantitated using Bio-Rad Quantity One Gel Doc version 4.6.9 software. All cultures were performed in triplicate and each miRNA experiment was repeated three times.

### Soft agar colony formation assay

Cells were trypsinized, resuspended in RPMI 1640 media containing 0.4% agarose and 10% FBS and overlaid onto a bottom layer of solidified 0.8% agarose in RPMI media 1640 containing 5% FBS, at concentrations of 3x10^3^ cells per well in 12-well plates, and incubated for 3 weeks. Colonies were stained with 0.05% crystal violet, photographed, and quantified.

### Endothelial cell morphogenesis assay: tubule formation

Human umbilical vein endothelial cells (HUVEC) grown to ~70% confluence were trypsinized, counted, and seeded with various conditioned media at 48,000 cells per well in 24-well plates coated with 300 µl of Matrigel (BD Biosciences, Cat No. 354234). These cells were periodically observed by microscope as they differentiated into capillary-like tubule structures. After 6 h, cells were stained with Hematoxylin & Eosin and photographs were taken with the microscope. The average number of tubules was calculated from examination of three separate microscopic fields (200X) and representative photographs were obtained.

### *In Vitro* scratch assay

To measure cell migration *in vitro*, a confluent monolayer of cells in a 6-well plate was scraped with a 200 µl pipet tip then incubated and observed microscopically from 0 to 72 h. Wound closure was calculated by measuring the remaining space in the microscopic images.

### Immunoblotting

Cells (5X10^5^) were collected at various time points, and then washed with ice-cold PBS twice before adding lysis buffer (M-PER Mammalian Protein Extraction reagent, Thermo Scientific, 78501) and cocktail inhibitor (5 µg/ml, Sigma, P8340). Equal amounts of protein were loaded into each well and separated by NuPAGE 4-12% Bis-Tris gel, followed by transfer onto PVDF-membranes (Bio-Rad, 162-0177). Membranes were blocked with 5% non-fat milk in PBS-T for 1 h at room temperature (RT). The blots were then incubated with antibodies against LC3 I &II. for 1 h at 4°C. Goat anti-rabbit IgG secondary antibody (1:5000; Santa Cruz Biotechnology, sc-2004) was then incubated for 45 min at RT. Immunoblots were developed by using the chemiluminescence detection system (PerkinElmer, NEL105) and autoradiography was performed. β-actin was used as a loading control.

### Autophagy assay

Parental, CDDP-R, Vin-R and Rad-R BE(2)-C cells were transfected with 3 µg of EGFP-LC3 expression plasmid (a gift from Dr. Noboru Mizushima) using Lipofectamine 2000 (Invitrogen, 11668). The fluorescence of EGFP-LC3 was observed using immunofluorescence microscopy. Cells with punctate GFP signals were counted as autophagic cells based on characteristic lysosomal localization of LC3 protein during autophagy [34]. Punctate GFP cells were quantified by randomly selecting three separated 100× fields and counting the number of punctate GFP cells per field. The percent of punctate GFP cells per total GFP-transfected cells was calculated and experiments were conducted in triplicate.

### Statistical analysis

All Scoring indexes are shown as mean value ± SEM from at least three independent experiments. Immunoblot scans are representative of three independent experiments. Statistical analysis was performed using a Student’s t-test. A *p* value < 0.05 was considered to be statistically significant.
